# *Haemonchus contortus* Transthyretin-Like Protein TTR-31 Plays Roles in Post-Embryonic Larval Development and Potentially Apoptosis of Germ Cells

**DOI:** 10.3389/fcell.2021.753667

**Published:** 2021-11-03

**Authors:** Hengzhi Shi, Xiaocui Huang, Xueqiu Chen, Yi Yang, Fei Wu, Chaoqun Yao, Guangxu Ma, Aifang Du

**Affiliations:** ^1^ College of Animal Sciences, Zhejiang Provincial Key Laboratory of Preventive Veterinary Medicine, Institute of Preventive Veterinary Medicine, Zhejiang University, Hangzhou, China; ^2^ Department of Biomedical Sciences and One Health Center for Zoonoses and Tropical Veterinary Medicine, Ross University School of Veterinary Medicine, Basseterre, Saint Kitts and Nevis

**Keywords:** *Haemonchus contortus*, transthyretin-like protein, post-embryonic larval development, apoptosis, phagocytosis

## Abstract

Transthyretin (TTR)-like proteins play multi-function roles in nematode and are important component of excretory/secretory product in *Haemonchus contortus*. In this study, we functionally characterised a secretory transthyretin-like protein in the barber’s pole worm *H. contortus*. A full-length of transthyretin-like protein-coding gene (*Hc-ttr-31*) was identified in this parasitic nematode, representing a counterpart of *Ce-ttr-31* in *Caenorhabditis elegans*. High transcriptional levels of *Hc-ttr-31* were detected in the egg and early larval stages of *H. contortus*, with the lowest level measured in the adult stage, indicating a decreased transcriptional pattern of this gene during nematode development. Localisation analysis indicated a secretion of TTR-31 from the intestine to the gonad, suggesting additional roles of *Hc-ttr-31* in nematode reproduction. Expression of *Hc-ttr-31* and *Ce-ttr-31* in *C. elegans* did not show marked influence on the nematode development and reproduction, whereas *Hc-ttr-31* RNA interference-mediated gene knockdown of *Ce-ttr-31* shortened the lifespan, decreased the brood size, slowed the pumping rate and inhibited the growth of treated worms. Particularly, gene knockdown of *Hc-ttr-31* in *C. elegans* was linked to activated apoptosis signalling pathway, increased general reactive oxygen species (ROS) level, apoptotic germ cells and facultative vivipary phenotype, as well as suppressed germ cell removal signalling pathways. Taken together, *Hc-ttr-31* appears to play roles in regulating post-embryonic larval development, and potentially in protecting gonad from oxidative stress and mediating engulfment of apoptotic germ cells. A better knowledge of these aspects should contribute to a better understanding of the developmental biology of *H. contortus* and a discovery of potential targets against this and related parasitic worms.

## Introduction

The strongylid nematode *Haemonchus contortus* (also known as barber’s pole worm) is one of the most important parasitic nematodes of sheep, goats and other ruminants (Chilton et al., 2006). It feeds on blood in the abomasa of small ruminants and causes the parasitic disease haemonchosis characterised by anemia, hemorrhagic gastritis, oedema and associated complications (Zajac and Garza., 2020). This parasitic disease affects hundreds of millions of livestock animals, causing billions of dollars of losses to livestock husbandry globally (Roeber et al., 2013; Wang et al., 2017; Zajac and Garza., 2020). Current control strategies against *H. contortus* infection in ruminants rely heavily on anthelmintic chemotherapy, especially in the absence of efficient alternative methods in many countries (Blanchard et al., 2018). However, frequent and indiscriminate administration of anthelmintics has led to the development of drug resistance worldwide (Arsenopoulos et al., 2021), including drugs recently introduced into the market (Mederos et al., 2014; Niciura et al., 2019). Although the commercial vaccine Barbervax^®^ against *H. contortus* has been registered for use in lambs in some countries (Teixeira et al., 2019; Kebeta et al., 2020), effective control of *H. contortus* and haemonchosis remains a major challenge globally. Revealing resistance mechanisms and discovering novel drug/vaccine targets are current priorities in the research field of *H. contortus*, which should be preferably based on a deep understanding of this important parasitic nematode at the molecular level (Wang et al., 2017; Ma et al., 2020).

Extensive studies on the excretory/secretory products (ESP) of parasitic nematodes (Hewitson et al., 2011; Vanhamme et al., 2020) have indicated a range of molecules that might play important roles at the host-parasite interface and are of potential value as vaccine targets. For instance, hundreds of ESPs of *H. contortus* have been identified by using advanced transcriptomic and proteomic tools, including proteolytic enzymes, glycoside hydrolases, C-type lectins, SCP/TAPS and transthyretin (TTR)-like proteins (Yatsuda et al., 2003; Gadahi et al., 2016; Wang et al., 2019). Particularly, TTR-like proteins have been consistently identified in the previous studies, which have been proposed to play a role in the developmental transition from the free-living stage to the parasitic stage of *H. contortus* as well as in the host-parasite interactions (see Cantacessi et al., 2010; Laing et al., 2013; Britton et al., 2016; Wang et al., 2019). In particularly, although it was proposed that TTR proteins play various roles in regulating apoptosis, modulating host immune responses and degenerative maladies in a range of organisms (Wang et al., 2010; Ankarcrona et al., 2016; Lin et al., 2016), few molecules have been studied at the molecular level in free-living or parasitic worms (Wang et al., 2010; Offenburger et al., 2018). Little is known about the biological function of TTR-like proteins in *H. contortus* and related parasitic nematodes of socioeconomic importance.

A recent proteomic study has confirmed 15 excreted/secreted TTR-like proteins in the parasitic stages of *H. contortus* (Wang et al., 2019), suggesting their roles in the key biological processes (e.g., development, reproduction and parasitism) of this important parasite. In this study, we elected to characterise one gene homologue (*Hc-ttr-31*) of *C. elegans* *ttr-31* that exhibited strong phenotypic changes in previous genome-wide RNA interference (RNAi) studies. Real-time quantitative PCR (qRT-PCR) was used to analyse the developmental transcription of *ttr-31* in *H. contortus* and *C. elegans*. Spatial expression of *Hc-ttr-31* was assessed in the infective third-stage larvae (L3s), fourth-stage larvae (L4s) and adults of *H. contortus* by indirect immunofluorescence, and in *C. elegans* N2 strain by transgenesis. Gene knockdown was conducted to investigate the functional roles of *Hc-ttr-31* in the development and reproduction as well as apoptosis of this important parasitic nematode.

## Materials and Methods

### Propagation of *H. contortus* and *C. elegans*

Adult worms of *H. contortus* (ZJ strain) were collected from a slaughterhouse in Jiaxing, Zhejiang, China. Female adult worms were dissected to collect eggs under a dissecting microscope (Motic, China). Eggs were cultured at 28°C for 7 days to obtain the L3s. Three helminth-free lambs (Hu sheep, 6 months old) were experimentally infected with ∼8000 L3s of *H. contortus* as described previously (Zhang et al., 2018). Faecal examination was performed to confirm the establishment of infection. Eggs of *H. contortus* were collected by flotation using saturated NaCl solution (Cox and Todd, 1962). The first-stage larvae (L1s), second-stage larvae (L2s) and L3s of *H. contortus* were obtained by incubating faecal samples at 28°C for 1, 3 and 7 days, respectively. L4s and adult worms were collected from the abomasum of euthanised lambs at 9 and 45 days after infection, washed in phosphate-buffered saline (PBS, pH7.4) and temporarily maintained in DMEM (Thermo Fisher Scientific, United States) with 10% FBS (Biological Industries, Israel) at 37°C (Shi et al., 2021).

The free-living nematode *C. elegans* N2 strain was maintained on nematode growth medium (NGM) agar plates at 20°C (Stiernagle, 2006). NGM agar plates were supplied with *Escherichia coli* OP50 or HT115 mutant bacteria as a food source.

### Extraction of Deoxyribo Nucleic Acid and Ribo Nucleic Acid

Genomic DNA (gDNA) was extracted from snap-frozen adult worms of *H. contortus* using a small-scale DNA extraction kit (Takara Bio, Japan) according to the supplier’s instructions. Total RNA was extracted from eggs, L1s, L2s, L3s, L4s and adult worms of *H. contortus* using Trizol reagent (Invitrogen, United States). The first strand cDNA was synthesised from the total RNA using a first-strand cDNA synthesis kit (Toyobo, Japan). Extracted gDNA and synthesised cDNA were stored at −80°C until use.

### Cloning and Characterization

The sequence of *C. elegans ttr-31* (WormBase ID: WBGene00010225) was used to identify the gene homologue of *H. contortus* (designated as *Hc-ttr-31*) from the Sanger Helminths Database (https://www.sanger.ac.uk/resources/downloads/helminths/haemonchus-contortus.html). Based on the available transcript of *Hc-ttr-31*, primers (Supplementary Table S1) were designed using Primer Premier 5 (PREMIER Biosoft International, United States) to amplify the coding region of this gene. The amplification reaction mix contained 2.5 μL 10 × PCR buffer, 0.5 μL LA Taq (Takara Bio), 2 μL of 2.5 mM dNTPs, 2.0 μL of primer pair (10 μM), 1 μL gDNA of *H. contortus* and 17 μL of molecular grade water. The thermocycle program comprised of 94°C for 15 s, 62°C for 30 s and 72°C for 30 s. PCR amplification was also performed using cDNA as a template to obtain the transcript of *Hc-ttr-31*. PCR products were purified, ligated to PMD-19T (Takara Bio) and sequenced in both directions. Based on the obtained nucleotide acid sequence, exons and introns of *Hc-ttr-31* were predicted by the “GT-AG” rule using DNASTAR (Version 7.1.0) (Breathnach and Chambon, 1981), with evidence from the obtained transcript sequences. Compara Gene Tree analysis and reciprocal blastp searching were performed based on resources available at WormBase ParaSite (https://parasite.wormbase.org/index.html). Functional domain architecture of the inferred proteins was predicted based on the InterPro database (Mitchell et al., 2018).

### Quantitative Real-Time Polymerase Chain Reaction

Transcriptions of *Hc-ttr-31* in all developmental stages (egg, L1, L2, L3, L4 and adult) of *H. contortus* were determined using Real-time SYBR Green Mix reagent (Toyobo) and a CFX96 Real-time PCR System (Bio-Rad, United States). qRT-PCR was also performed to assess the transcriptional level of *ttr-31* in different developmental stages of *C. elegans*. The thermocycle program was 95°C for 10 min followed by 40 cycles of 95°C for 15 s, 60°C for 15 s and 72°C for 15 s. *Actin* (*act-1*) and *β-tubulin* were used as the internal controls for *C. elegans* and *H. contortus*, respectively. Transcriptional levels of key signalling components (i.e., *ced-1*, *ced-4*, *ced-6*, *ced-7*, *ced-9*, *egl-1*, *ina-1, nrf-5*, *par-1*, and *ttr-52*) involved in apoptosis and phagocytosis of apoptotic cells (Reddien and Horvitz, 2004; Wang et al., 2010; Palmisano and Meléndez, 2019) were also assessed. Primer sets used in this section can be found in Supplementary Table S1. Three replicates were included and performed independently. Transcriptional changes of genes were analysed using a 2^-∆∆Ct^ method and presented as mean ± standard error of mean (SEM). Statistical analysis was conducted using a one-way ANOVA (*p* < 0.05).

### Prokaryotic Expression and Preparation of Polyclonal Antibodies

*Hc-ttr-31* was subcloned into pET30a vectors for prokaryotic expression of the recombinant protein r*Hc*-TTR-31. In brief, the pET30a*-Hc-trr-31* plasmids were transformed into *E. coli* BL 21 (DE3), which was induced by 0.5 mM isopropyl-*β*-d-1-thiogalactopyranoside (IPTG) at 37°C for 8 h. Bacteria were harvested by a centrifugation at 8,000 × g at 4°C for 5 min, suspended in 50 mM potassium phosphate (pH7.4), and lysed by sonication. The supernatant was separated by a centrifugation at 12,000 × g for 10 min at 4°C, filtered with a 0.22 μm filter (Merck Millipore, United States), then incubated with Ni-NTA resin column (Thermo Fisher Scientific) for 30 min. After washing with 20 mM imidazole, recombinant protein r*Hc*-TTR-31 was eluted with 250 mM imidazole from the column. Purified protein was assessed by SDS-PAGE and a Bradford kit (Fdbio Science, China) using bovine serum albumin (BSA) as standard.

A New Zealand rabbit was immunised with the r*Hc*-TTR-31 to generate polyclonal antibodies against the recombinant protein (Shi et al., 2021). In brief, the rabbit was subcutaneously injected with the r*Hc*-TTR-31 and Freund’s Complete Adjuvant (Sigma-Aldrich, United States). Two boosting shots were conducted with the r*Hc*-TTR-31 and Incomplete Fraud’s Adjuvant in a 2-week interval. Antiserum was collected 10 days after the final immunisation. Western blotting was performed using the anti-6 × His tag polyclonal antibodies (1:2,000; Proteintech, United States) and the generated anti-r*Hc*-TTR-31 polyclonal antibodies to determine the specificity of antibodies.

### Indirect Fluorescence Immunohistochemistry

Immunolocalisation of target protein was performed as described previously (Riou et al., 2005; Shi et al., 2021). Briefly, L3s of *H. contortus* were exsheathed by incubation with 0.15% sodium hypochlorite for 30 min (Ding et al., 2017); the exsheathed L3s (xL3s), L4s and adults were washed in PBS (pH7.4), fixed in 4% paraformaldehyde (Rothwell and Sangster, 1993) overnight, and then dehydrated. L4s and adults were further embedded in paraffin, sectioned at 5 μm in thickness, dried at 60°C overnight and deparaffinized with xylene and dehydrated as described previously (Qu et al., 2014). Sliced L4s and adults as well as xL3s of *H. contortus* were incubted in 1% BSA for 2 h, washed twice in PBS, and then incubated with 1:500 diluted anti-*Hc*-TTR-31 polyclonal antibodies at 4°C overnight. After washing, slides were incubated with goat anti-rabbit IgG (Invitrogen) at 1:1,000 dilution at 37°C for 1 h. Pre-immunisation serum from the same animal was used as a negative control. Fluorescence was detected with a Zeiss LSM710 laser confocal microscope (Zeiss Microscopy, Germany).

### Transgenic Expression of *Hc-ttr-31* in *C. elegans*

To confirm the spatial distribution of *Hc*-TTR-31, transgenesis of *Hc-ttr-31* was conducted in *C. elegans* (Huang et al., 2021). Briefly, the promoter sequence of *Ce-ttr-31* was amplified, ligated to the PMD-18T vector and subcloned into pPD95.67 plasmids (*Ce-ttr-31p::gfp*), with the *Hc-ttr-31* coding sequence inserted into the multiple cloning site (*Ce-ttr-31p::Hc-ttr-31::gfp*). Transgenic expression of *Hc-ttr-31* in *C. elegans* N2 strain was performed by micro-injection with the *Ce-ttr-31p::Hc-ttr-31::gfp* (Mello et al., 1991). A *Ce-ttr-31p::Ce-ttr-31::gfp* was used as a reference control. Considering the homology of *Hc-ttr-31* and *Ce-ttr-31*, transgenesis of *Hc-ttr-31* should result in an over-expression of TTR-31 in the treated *C. elegans*. To explore the effects of exogenous *Hc*-TTR-31 in *C. elegans*, transgenic worms were mounted on 2% agar pads containing 1% sodium azide (Solarbio, China) and imaged using a Zeiss LSM710 laser confocal microscope (Zeiss Microscopy).

### RNA Interference

To explore the function of *Hc-ttr-31* in *H. contortus*, *Hc-ttr-31* RNAi-mediated gene knockdown of its orthologue *Ce-ttr-31* was performed in *C. elegans*, using a method described previously (Kwon et al., 2010; Yan et al., 2014). In brief, *Hc-ttr-31* was cloned into the plasmid L4440, which was then transformed into *E. coli* HT115 (DE3), with *Ce-ttr-31* and empty L4440 vectors used as positive and negative controls, respectively. NGM plates containing 0.1 mM IPTG were seeded with the transformed HT115 and placed at room temperature for 5 days prior to RNAi assay. Worms of *C. elegans* N2 strain were synchronized and laid on standard NGM plates supplemented with OP50 (Kamath and Ahringer, 2003). The worms were washed off the plates after 30 h (i.e., L4 stage) and then centrifuged at 1,000 ×g for 5 min. From which, 20 worms were inoculated onto NGM plates with the transformed HT115, and incubated at 25°C for 3 days. Following transcriptional examinations of *Hc-ttr-31* and *Ce-ttr-31* by qRT-PCR, phenotypic changes of worms were assessed in aspects of brood size, pumping rate, body length and body width (see Wong et al., 1995; Mörck and Pilon, 2006; Papaevgeniou et al., 2019). Three replicates were performed for the RNAi and phenotypic assays. For the analysis of facultative vivipary phenotype, worms were checked using a dissecting microscope (Motic) and the ratio of bagging worms was calculated.

### Apoptosis Assay

To check whether *Hc-ttr-31* plays a role in the apoptosis, the number of apoptotic germ cells in the RNAi-treated worms was determined using a method described elsewhere (Kelly et al., 2000) with some modifications according to manufacturer’s instruction. Briefly, ∼ 100 synchronised adult worms were subjected to RNAi treatment for 36 h and then washed in M9 buffer for 5 times; Worms were suspended in 0.5 ml M9 buffer and stained with acridine orange (20 μg/ml; Solarbio) for 1.0 h; Stained worms were then transferred onto NGM plates seeded with OP50 for 1.5 h for recovery. Recovered worms were kept in 1.0% sodium azide solution at the centre of slide and examined under a Zeiss LSM710 laser confocal microscope (Zeiss Microscopy).

### Measurement of Reactive Oxygen Species

Production of general ROS in RNAi-treated worms was determined using 2′, 7′-dichlorodihydrofluorescein diacetate (DCFH-DA) (Beyotime, China) based on the method reported elsewhere (Zhu et al., 2010; Guo et al., 2016). In brief, about 5,000 treated/untreated worms were sonicated in PBS, and the supernatant was collected after centrifugation at 15,000 × g for 45 min. Bradford protein assay kit (Fdbio Science) was performed to determine protein concentration. The lysates were incubated with 300 μM DCFH-DA in black-walled microtiter plate (Corning Incorporated, United States) at 37°C for 3 h. Rosup was used as a positive control in the production of reactive oxygen. Fluorescence intensity was measured using a Synergy H1 hybrid multimode microplate reader (BioTek, United States).

### Statistical Analysis

At least three technical replicates were included in each assay and each experiment was repeated three times. Data are presented as mean ± standard error of mean (SEM). One-way ANOVA with Dunnett post-hoc test was performed using Excel 2016 (Microsoft, United States) and GraphPad Prism 5 (GraphPad Software, United States). *p* < 0.05 was considered statistically significant.

## Results

### cDNA-Confirmed *Hc-ttr-31* is a 1-to-1 Orthologue of *Ce-ttr-31*

**FIGURE 1:**
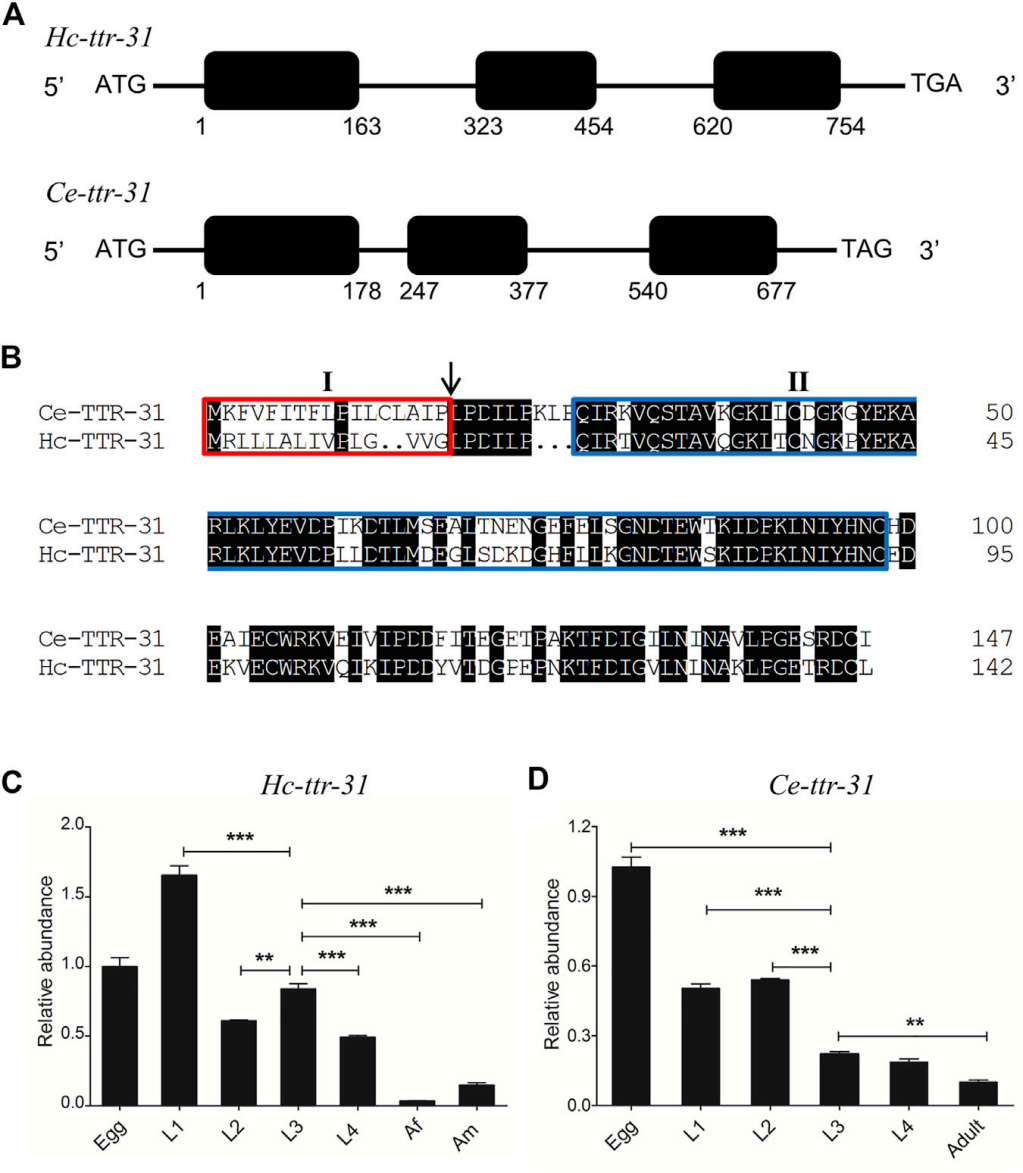
Sequence and transcription analyses of *ttr-31* in *Haemonchus contortus* and *Caenorhabditis elegans*. **(A)** Gene structures of *Hc-ttr-31* and *Ce-ttr-31*. Black boxes and the horizontal lines represent exons and introns, respectively. The numbers below the black boxes indicates the boundaries of exons. **(B)** Pair-wise sequence alignment of the predicted TTR-31 of *C. elegans* and *H. contortus*. Identical residues are shaded in black. The red box (I) indicates the predicted secretory signal, the arrow points the putative cleavage sites and the blue box (II) shows the transthyretin domain. Transcriptional patterns of *Hc-ttr-31* **(C)** and *Ce-ttr-31* **(D)** in different developmental stages of *H. contortus* and *C. elegans*. Egg, first (L1), second (L2), third (L3), fourth (L4) larval, female adult (Af) and male adult (Am) stages are indicated. *p < 0.05; **p < 0.01; ***p < 0.001.

*Hc-ttr-31* comprised three exons, encoding a transcript of 432 nt in length (GenBank accession number MW013314; Figure 1A), representing a predicted gene locus (hcontortus_chr2_Celeg_TT_arrow_pilon: 30031855–30032608) in the recently updated chromosome-level genome assembly of *H. contortus* (MHCO3ISE; WBPS15). The cDNA-confirmed gene model of *Hc-ttr-31* was similar to that of *Ce-ttr-31* in the free-living model organism *C. elegans* (Figure 1A), and was predicted a 1-to-1 orthologue of *Ce-ttr-31*. The inferred protein *Hc*-TTR-31 was 143 aa in length, contained a signal peptide (SignalP-noTM) and a transthyretin-related family domain, showing a 65% similarity to the amino acid sequence deduced from *Ce-ttr-31* (Figure 1B).

### *Hc-ttr-31* Highly Transcribes in all Larval Stages but Not in Adult Stage

Different transcriptional levels of *Hc-ttr-31* were detected among the developmental stages of *H. contortus*. Specifically, *Hc-ttr-31* was highly transcribed in the egg and larval (L1, L2, L3 and L4) stages of *H. contortus*, with the highest level detected in the L1 stage and the lowest in the female adult stage (Figure 1C), showing a decreasing transcriptional pattern of *Hc-ttr-31* during the development of this parasitic nematode. A similar developmental transcription of *Ce-ttr-31* was also found in *C. elegans* (Figure 1D). Differently, the highest transcriptional level of *Ce-ttr-31* was detected in the egg of the free-living nematode (Figure 1D).

### *Hc*-TTR-31 is Localised in the Intestine and Gonad of *H. contortus*

**FIGURE 2:**
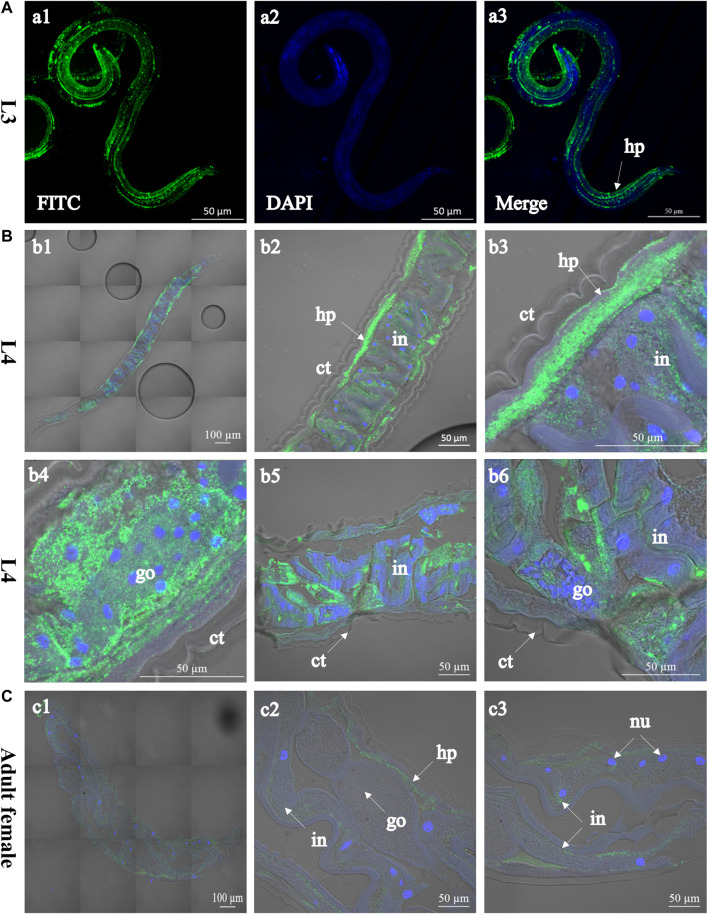
Immunolocalisation of *Hc*-TTR-31 in *Haemonchus contortus*. Green fluorescence shows the distribution of *Hc*-TTR-31 in the third- **(A)** and fourth stage larvae **(B)**, and adult stage (female) **(C)** of *H. contortus*. *Hc*-TTR-31 is probed with rabbit anti-r*Hc*-TTR-31 polyclonal antibodies followed by fluorescein (FITC) conjugated-goat anti-rabbit IgG as secondary antibody. Nuclei are stained with 4′,6-diamidino-2-phenylindole (DAPI). ct: cuticle, go: gonad, in: intestine, hp: hypodermis, nu: nucleus. Scale bar: 50 μm or 100 μm.

Recombinant *Hc-*TTR-31 (r*Hc-*TTR-31) was successfully expressed in *E. coli* BL21 (DE3) and was recognised by the anti-6×His tag polyclonal antibodies (Supplementary Figure S1). Using the recombinant protein, polyclonal antibodies against r*Hc-*TTR-31 were generated and used to detect the native *Hc-*TTR-31 from the crude protein extract of *H. contortus* (Supplementary Figure S1A), facilitating immunolocalization of native *Hc-*TTR-31 in the L3s, L4s, and adults of this parasite (Figure 2 and Supplementary Figure S2). Specifically, predominant cephalic, cuticular/muscular and intestinal distributions of *Hc-*TTR-31 were observed in the xL3s of *H. contortus*, with punctate expression detected in the head and tail regions (Figure 2A). Muscular and intestinal distributions of *Hc-*TTR-31 were also found in L4s (Figure 2B) and adults (Figure 2C) of this parasitic nematode. Differently, the fluorescent intensities observed in the L4s and adults were lower than that in the xL3s of *H. contortus* (Figures 2B,C), which was in accordance with the decreasing transcriptional pattern of *Hc-ttr-31* during the development of this parasite.

### Expression of *Ce*-TTR-31 and *Hc*-TTR-31 Prolonged the Lifespan of *C. elegans*

**FIGURE 3:**
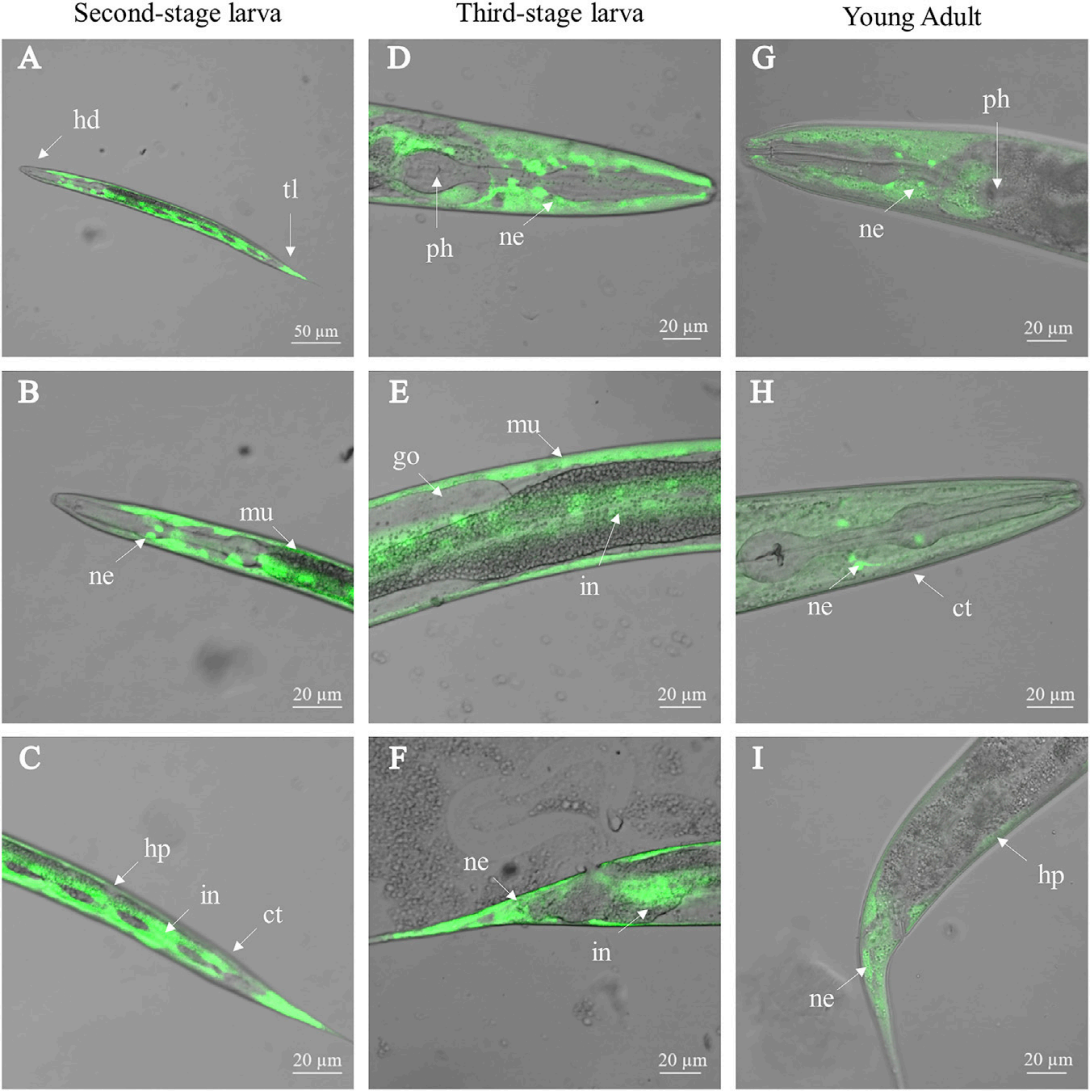
Activity of *Ce-ttr-31* promoter in *Caenorhabditis elegans*. *Ce-ttr-31p*:*gfp* is expressed in the second-stage larva **(A–C)**, third-stage larva **(D–F)** and adult **(G–I)** of *C. elegans*. Activities of *Ce-ttr-31* promoter in neurons (ne), muscle layer (mu), intestine (in) and hypodermis (hp) are indicated. ct: cuticle, go: gonad, hd: head, ph: pharynx, tl: tail. Scale bar: 20 μm.

**FIGURE 4:**
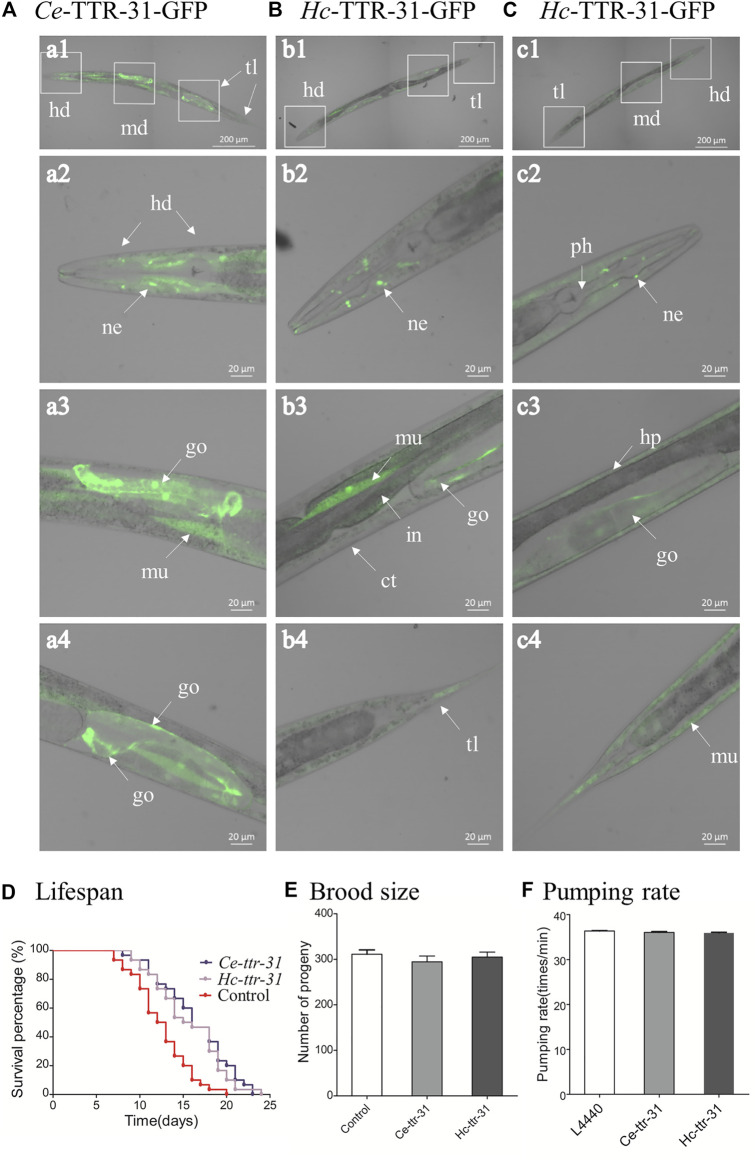
Transgenic expression of *Hc-ttr-31* in *Caenorhabditis elegans*. Green fluorescence protein-fused *Ce-*TTR-31 **(A)** and *Hc*-TTR-31 **(B,C)** were expressed in adult worms of *C. elegans* by microinjection of pPD95.67-*Ce-ttr-31p*:*gfp* plasmids. Protein expression in neurons (ne), gonad (go), muscle layer (mu) and hypodermis (hp) are indicated. The influences of overexpressed TTR-31 on lifespan **(D)**, brood size **(E)** and pumping rate **(F)** in transgenic worms are indicated. Data are presented as mean ± SEM (*n* = 30, 10, 10). ct: cuticle, hd: head, in: intestine, md: middle, ph: pharynx, tl: tail. Scale bar: 20 μm or 200 μm.

Driven by the promoter of *Ce-ttr-31* (*Ce-ttr-31p*), GFP was expressed in the intestine, muscle, and neurons in the pharynx and tail regions of *C. elegans* (Figure 3), suggesting functional activities of the promoter in these tissues. Therefore, by microinjection of the *Ce-ttr-31p::Ce-ttr-31::gfp* and *Ce-ttr-31p::Hc-ttr-31::gfp* plasmids, GFP-fused proteins were expressed in the transgenic worms. Specifically, the *Ce*-TTR-31-GFP fusion protein was observed in the pharyngeal neurons, muscle, and the U-shape gonad arms, but not detected in the intestine of adult worms (Figure 4A). A similar protein distribution was also found for the *Hc*-TTR-31-GFP fusion protein in the transgenic *C. elegans*, including pharyngeal neurons, gonad and musculature (Figures 4B,C).

Microinjection of *Ce-ttr-31p::Ce-ttr-31::gfp*/*Ce-ttr-31p::Hc-ttr-31::gfp* in *C. elegans* N2 strain resulted in expression of *Ce*-TTR-31 and expression of both *Ce*-TTR-31 and *Hc*-TTR-31 in the transgenic worms. Compared with the non-transgenic *C. elegans* N2 strain, expression of *Ce*-TTR-31 prolonged the lifespan of transgenic worms (Figure 4D), but did not show any significant (*p* > 0.05) effect on brood size (Figure 4E) or pumping rate (Figure 4F) of the transgenic worms. Expression of both *Ce*-TTR-31 and *Hc*-TTR-31 in *C. elegans* N2 strain showed similar effects to the expression of *Ce*-TTR-31 (Figures 4D–F).

### Downregulation of *ttr-31* Leads to Facultative Vivipary Phenotype in *C. elegans*

**FIGURE 5:**
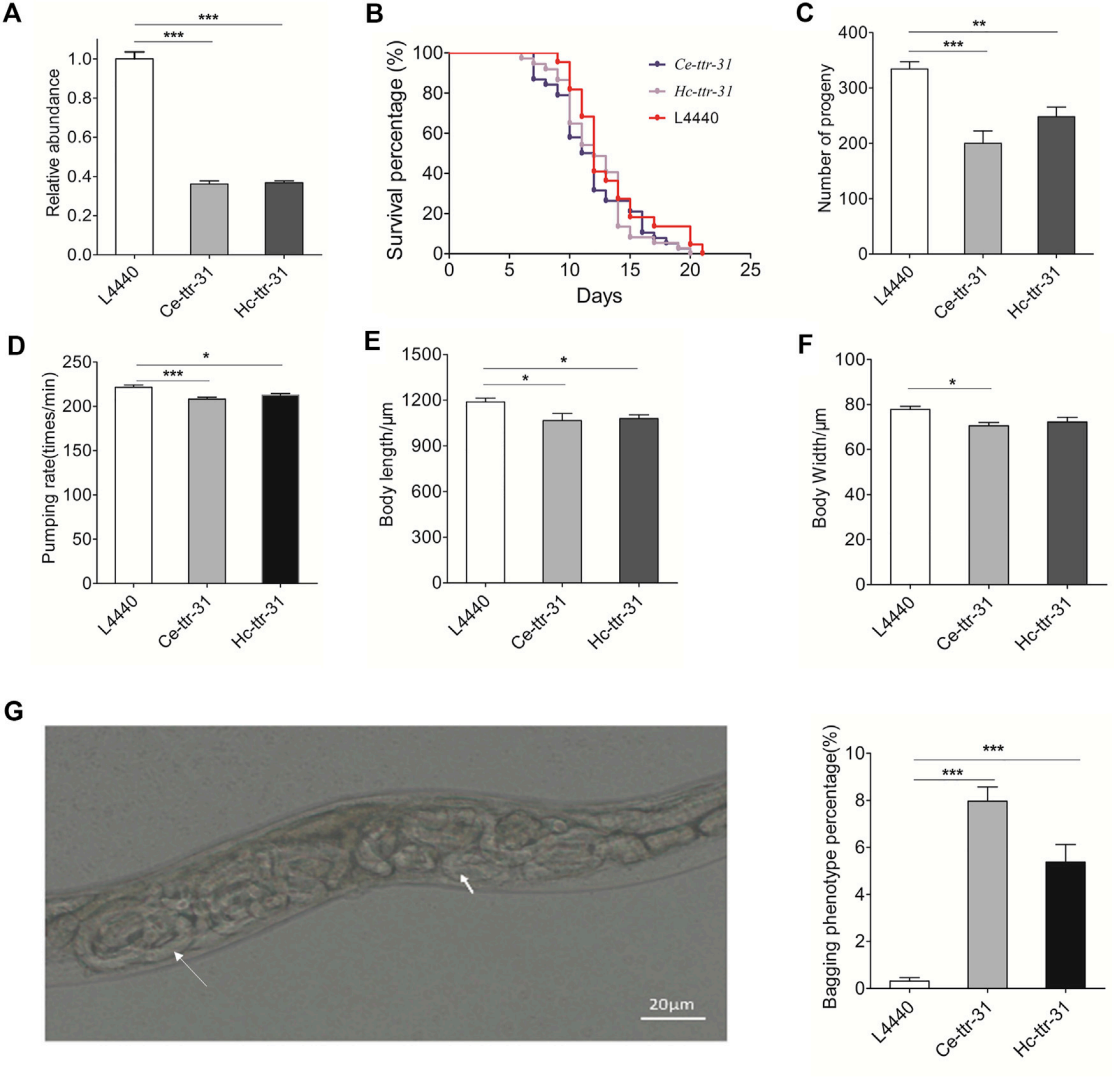
*Hc-ttr-31* RNAi mediated gene knockdown of *Ce-ttr-31* in *Caenorhabditis elegans*. **(A)** Transcriptional levels of *Ce-ttr-31* in treated worms. Effects of gene knockdown on the lifespan **(B)**, brood size **(C)**, pumping rate **(D)**, body length **(E)** and body width **(F)** are shown. Data are presented as mean ± SEM (*n* = 10). **(G)** Hatching *in vivo* phenotype and the percentage of bagging phenotype are shown. Data are presented as mean ± SEM (*n* = 6). *p < 0.05; **p < 0.01; ***p < 0.001.

To further explore the functional roles of *Hc-ttr-31*, gene knockdown was successfully conducted by feeding worms with HT115 bacteria that could express silencing RNAs targeting *Ce-ttr-31* or *Hc-ttr-31*. Specifically, compared with negative control, RNAi of *Ce-ttr-31* significantly reduced (>50%; *p <* 0.001) the transcriptional level of this gene in the treated worms (Figure 5A), shortened the lifespan of treated worms (Figure 5B), decreased the number of progeny (*p* < 0.001), and inhibited the pumping rate (*p* < 0.001) and growth (body length and width; *p* < 0.05) of the treated worms (Figures 5C–F). *Hc-ttr-31* RNAi mediated gene knock down of *Ce-ttr-31* also achieved effective gene silencing (>50%; *p <* 0.001) of *Ce-ttr-31* (Figure 5A), and resulted in similar effects on treated worms, such as shortened lifespan, and reduced brood size, pumping rate, body length and width (Figures 5B–F), implying functional conservation of *ttr-31* between *C. elegans* and *H. contortus*.

Notably, compared with the untreated *C. elegans*, effective knockdown of *Ce-ttr-31* and *Hc-ttr-31*-mediated knockdown of *Ce-ttr-31* led to significantly increased (8 and 5 fold; *p* < 0.001) facultative vivipary phenotype (i.e., hatching *in vivo* or “bagging”; Chen and Caswell-Chen, 2004) in treated worms (Figure 5G), suggesting involvement of TTR-31 in the post-embryonic development and reproduction processes.

### *Hc*-TTR-31 is Likely to Play a Role in the Apoptotic Germ Cell Removal

**FIGURE 6:**
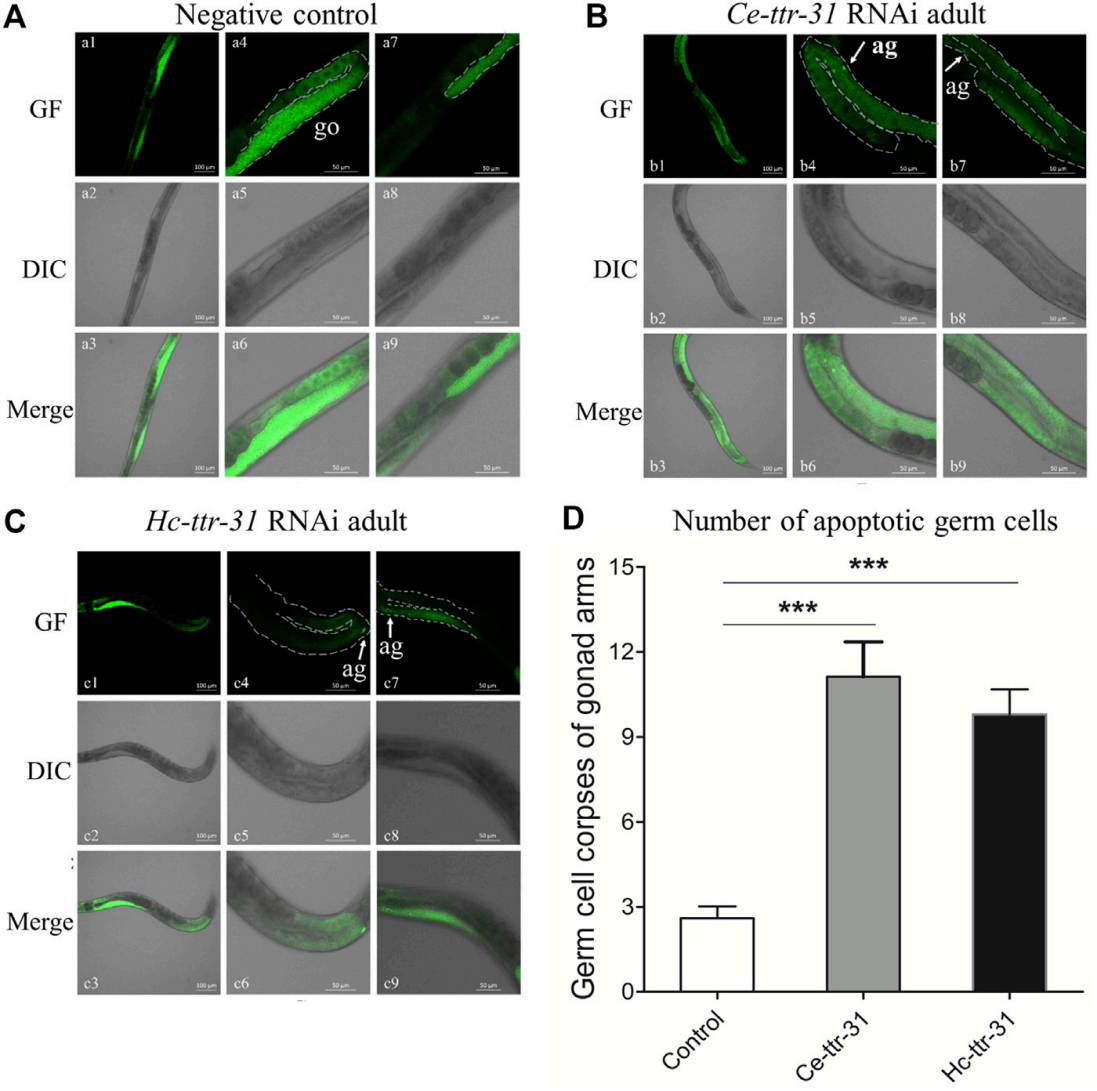
Effect of *ttr-31* RNA interference on the germ cells in *Caenorhabditis elegans*. Apoptotic germ cells in the negative control **(A)**, *Ce-ttr-31* **(B)** and *Hc-ttr-31* **(C)** RNAi-treated worms are indicated. Apoptotic germ cells (ag) are pointed by white arrows. **(A–C)** a1-a3 shows the whole gonad of *C. elegans* stained by acridine orange including two gonad arms (a4-a6 and a7-a9). b4-b6 and b7-b9 are two gonad arms of a worm in b1-b3. c4-c6 and c7-c9 are two gonad arms of a worm in c1-c3. **(D)** The number of apoptotic germ cells in the treated and untreated worms is shown. Data are presented as mean ± SEM (*n* = 15). GF: green fluorescence; DIC: differential interference contrast; Merge: GF merges with DIC. Scale bars: 50 μm ***p < 0.001.

**FIGURE 7:**
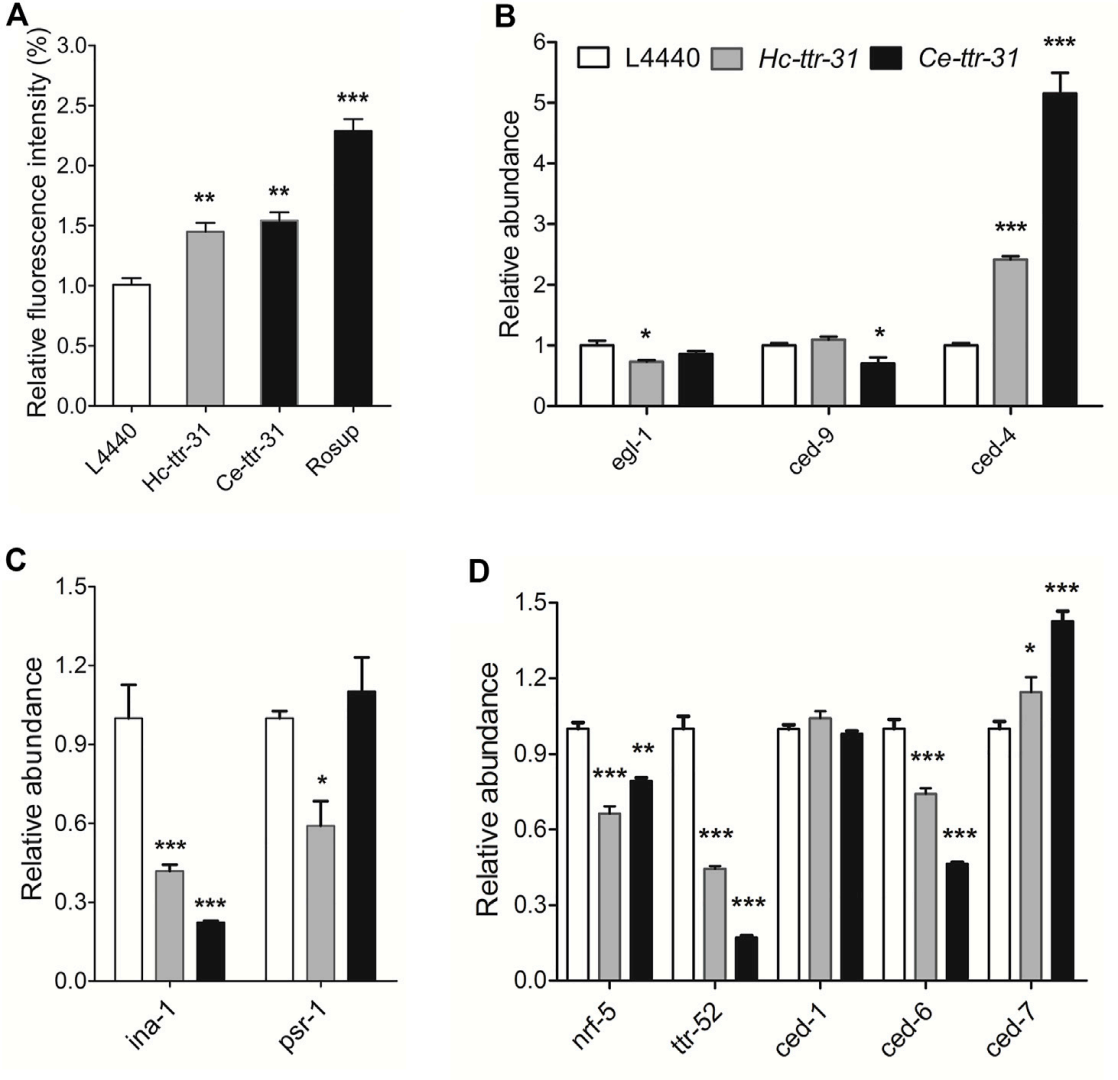
Effect of *ttr-31* knockdown on the apoptotic signalling pathway in *Caenorhabditis elegans*. **(A)** Relative levels of reactive oxygen species (ROS) in RNA interference (RNAi)-treated worms, negative and positive (Rosup) controls are shown. **(B–D)** Transcriptional levels of genes involved in apoptotic signalling pathways in RNAi-treated *C. elegans* worms. *ced-4*: *cell death abnormal 4*, *egl-1*: *egg-laying defective-1*, *ina-1*: *integrin-α*, *nrf-5*: *nose resistant to fluoxetine 5*, *psr-1*: *phosphatidyl serine receptor-1*. *p < 0.05; **p < 0.01; ***p < 0.001.

By staining with acridine orange, apoptotic cells were observed (brighter than the normal ones due to pyknosis) in the gonad arms of the RNAi-treated *C. elegans* (Figure 6). Specifically, compared with negative control (Figure 6A), there was an increase (4.28 and 3.77 folds) of apoptotic germ cells in the gonad arms of *Ce-ttr-31* and *Hc*-*ttr-31* RNAi-treated worms (Figures 6B,C). In addition to the increased number of apoptotic germ cells, lower transcriptional level of *Ce-ttr-31* and *Hc*-*ttr-31* was linked to a significant increase of general ROS (*p* < 0.01) and transcriptional level of *ced-4* (*cell death abnormal 4*, encodes a key component in the apoptosis activation pathway) (*p* < 0.001) in RNAi-treated worms (Figure 7A), suggesting activated apoptosis and involvement of *ttr-31* in apoptosis.

As the homologous gene *ttr-52* (encodes an extracellular phosphatidylserine-binding protein on the apoptotic cell) was reported to be required for cell corpse engulfment (Wang et al., 2010), the function of *ttr-31* in apoptosis or clearance of cell corpse was determined by exploring the transcriptional alterations of signalling components involved in phagocytosis of apoptotic cells (Figures 7B–D). Specifically, compared with untreated worms, significant lower transcriptions were detected for *ina-1* (encodes an engulfment receptor integrin-1 that functions upstream of CED-2/CED-5/CED-10/CED-12 apoptotic cell removal signalling pathway) (*p* < 0.001) (Figure 7C), and *nrf-5* (encodes a secreted lipid-binding protein) (*p* < 0.01), *ttr-52* (*p* < 0.001) and *ced-6* (encodes CED-6, a key factor in CED-1/CED-6/CED-7 cell corpse engulfment pathway) (*p* < 0.001) in both *Ce-ttr-31* and *Hc*-*ttr-31* RNAi-treated *C. elegans* (Figure 7D). Differently, a significant higher transcription of *ced-7* (encodes an ABC transporter that transfers phosphatidylserine from apoptotic cells to engulfing cells) (*p* < 0.05) was found in the treated worms (Figure 7C). Transcriptional changes of *egl-1* (*egg-laying defective-1*), *ced-9* (an orthologue of *bcl-2*), *psr-1* (encodes a phosphatidylserine-recognizing receptor) and *ced-1* (encodes a single-pass transmembrane protein that acts in engulfing cells to promote removal of apoptotic cells) were not consistent between *Ce-ttr-31* and *Hc*-*ttr-31* RNAi-treated worms (Figures 7B–D). In summary, *ttr-31* is likely to regulate and recognise the apoptotic cells.

## Discussion

The biological role of TTR-like proteins is not clear in parasitic nematodes, although such molecules have been commonly identified in the excretory/secretory products of parasitic worms. In this study, we reported the functional roles of *Hc-ttr-31* in a strongylid nematode *H. contortus*. We found that this gene was transcribed in all developmental stages of *H. contortus*, with a decreasing transcriptional pattern during the development from the egg to the adult stage. Immunolocalisation and transgenic expression analyses of *Hc-*TTR-31 indicated pharyngeal, muscular and gonad protein distributions of this protein. Gene knockdown of *Hc-ttr-31* in *C. elegans* was linked to an increase of ROS level and apoptotic germ cells, which resulted in a “worm bagging” phenotype, suggesting essential roles in development and reproduction of nematodes.

*Hc-ttr-31* is an orthologue to the *Ce-ttr-31* of the free-living nematode *C. elegans*. Although several TTR-like proteins or protein-coding genes have been reported in *H. contortus* (see Laing et al., 2013; Britton et al., 2016; Wang et al., 2019) and other parasitic nematodes (Parkinson et al., 2004; Saverwyns et al., 2008; Vieira et al., 2020), most of these molecules have not yet been identified and functionally characterised. In the current study, gene model validation, reciprocal homology searching, and domain architecture analysis confirmed the orthologous relationship between *Hc-ttr-31* and *Ce-ttr-31*. This statement was also supported by the similar transcriptional patterns and protein localisation of the two genes. In particular, RNAi targeting the transgenic *Hc-ttr-31* in *C. elegans* also resulted in efficient knockdown of *Ce-ttr-31* and the same phenotypes in transgenic worms, strongly suggesting sequence and functional conservation between the two genes. However, considering the nature of differences between the free-living nematode *C. elegans* and the parasitic nematode *H. contortus*, detailed biological exploration of *Hc-ttr-31* is still required.

It appears that *Hc-*TTR-31 was produced in the intestine and then secreted into the gonad of a worm. Given the extensive identification of TTR-like proteins in excretory/secretory products, it is interesting that transgenic expression of GFP (driven by the promoter of *Ce-ttr-31*) was observed in the intestine of *C. elegans*, whereas the fusion protein *Hc-*TTR-31-GFP was detected in the gonads of worms. It is likely that *Hc-*TTR-31 was synthesised in the intestinal cells, secreted into the body cavity, and then diffused to the gonad, which can be supported by the identification of signal peptide and the localisation of *Hc-*TTR-31 in *H. contortus*. Particularly, TTR-52 (a homologous to TTR-31) has been confirmed as a secretory protein that was found expressed in the intestine of *C. elegans* (Wang et al., 2010; Raiders et al., 2021). Additionally, TTRs are proteins commonly found in the serum and cerebrospinal fluid in mammals (Goodman, 1986; Herbert et al., 1986; Buxbaum and Reixach, 2009), suggesting the secretory nature of these proteins. However, it is still not clear whether there is a polarity of *Hc-*TTR-31 secretion (exclusive secretion into body cavity or intestine lumen), since it has been identified in the excretory/secretory products of *H. contortus* (Wang et al., 2019) and potentially other parasitic nematodes.

The secretory *Hc-*TTR-31 might play a role in nematode development, particularly post-embryonic larval development. This statement can be strongly supported by the transcriptional analysis, protein localisation and RNAi analysis in the current study. First, *Hc*-*ttr-31* was highly transcribed in the early developmental stages (e.g., egg, L1, L2 and L3) and then significantly downregulated when worms enter parasitic stages (e.g., L4 and adult) of *H. contortus*. More transcriptional evidence can be found in a previous study in which significant differences in transthyretin-like gene families were found between the free-living and the CO_2_-activated L3s of *H. contortus* (Cantacessi et al., 2010). Second, *Hc-*TTR-31 was consistently found in the pharyngeal neurons of *H. contortus* L3s and *C. elegans* larvae (i.e., L2s and L3s), whereas expression of both *Hc*-*ttr-31* and *Ce*-*ttr-31* in adult *C. elegans* extended the lifespan but did not influence the pumping rate and brood size of the transgenic worms. TTRs were also found in cerebrospinal fluid of mammals and mutations of these proteins may cause Alzheimer’s disease in human beings (Schwarzman et al., 1994; Sousa et al., 2007; Gião et al., 2020). So, it is very likely that *ttr-31* plays a role in signalling pathways, such as insulin/insulin-like growth factor 1 (insulin/IGF-1) and steroid hormone signalling (Murphy and Hu, 2013; Antebi, 2015), to regulate worm growth and lifespan. However, this statement still needs further exploration and verification at the molecular level. Third, *Hc*-*ttr-31* RNAi-mediated gene knockdown *Ce*-*ttr-31* resulted in slower pumping rate and growth as well as a significant increase of embryos in the utero and egg hatching *in vivo* in *C. elegans* (facultative vivipary; see Chen and Caswell-Chen, 2004). Post-embryonic development variant, larval arrest and slow growth have also been reported in the previous RNAi experiments of *C. elegans* (Kamath et al., 2003; Rual et al., 2004; Dalton and Curran, 2018). Therefore, it is clear that *Hc*-*ttr-31* also plays potential roles in regulating the post-embryonic development, larval growth and lifespan of *H. contortus*. Nevertheless, the mechanism of *ttr-31* underlying the developmental regulation of parasitic worms is still unclear and warrants further investigation.

In addition, *Hc-*TTR-31 might play dual roles in protecting germ cells from oxidative stress and mediating clearance of apoptotic cells. It has been reported that a transthyretin-like protein (TTR-52) was required to mediate recognition and clearance of apoptotic cells in *C. elegans* (Wang et al., 2010; Palmisano and Meléndez, 2019). In the current study, gene knockdown of *ttr-31* led to an increase of ROS production and increased number of apoptotic germ cells, as well as facultative vivipary (also known as worm bagging, a survival-enhancing response to stress; Chen and Caswell-Chen, 2004). Therefore, it is likely a relationship between low TTR-31 expression and oxidative stress which then causes DNA damage and apoptosis in the gonadal tissue of RNAi-treated worms (see Stergiou and Hengartner, 2004; Balaban et al., 2005; Hubbard and Schedl, 2019). Indeed, the functional role of another transthyretin-like protein (TTR-33) in protecting dopaminergic neurons from oxidative stress-induced degeneration has been reported in *C. elegans* (Offenburger et al., 2018). However, it is still plausible that dying cells might be recognised and cleared by phagocytes (phagocytosis) via TTR-31 (Lockshin and Zakeri, 2001; Reddien and Horvitz, 2004), as no apoptotic germ cell was found in the wild-type *C. elegans*. To further distinguish or confirm the involvement of TTR-31 in apoptosis and in apoptotic cell clearance, we assessed the transcriptional status of key signalling components involved in the apoptosis signalling pathways (see Savill and Fadok, 2000; Hochreiter-Hufford and Ravichandran, 2013; Palmisano and Meléndez, 2019). First, *ced-4* (a key component in the apoptosis activation pathway) was up-regulated in *ttr-31* RNAi treated worms, suggesting an initiation of apoptosis (see Stergiou and Hengartner, 2004; Hochreiter-Hufford and Ravichandran, 2013). Second, *ina-1* (an engulfment receptor functions upstream of CED-2/CED-5/CED-10/CED-12 apoptotic cell removal signalling pathway), *nrf-5* and *ttr-52* (molecules mediating engulfment signal into CED-1/CED-6/CED-7 pathway) were down-regulated in response to gene knockdown of *ttr-31* RNAi, suggesting a suppressed phagocytosis of apoptotic cells (Wang et al., 2003; Hsu and Wu, 2010; Zhang et al., 2012; Haley et al., 2018). Third, *ced-7* (an ABC transporter that transfers phosphatidylserine from apoptotic cells to engulfing cells; Wang et al., 2003) was found upregulated in *ttr-31* knock-down worms, which might be associated with the increased apoptotic germ cells. Therefore, *Hc-*TTR-31 is likely to play roles not only in protecting germ cells from oxidative stress-induced apoptosis and but also in mediating apoptotic germ cell clearance. However, this functional interpretation is based on results derived from heterologous expression of *Hc-ttr-31* and heterologous RNAi of *Ce-ttr-31* in *C. elegans*. A rescuing experiment of *Ce-ttr-31* loss-of-function by *Hc-ttr-31* in *C. elegans*, or direct gene knockout assay in *H. contortus* should be preferably conducted. Further investigations such as binding assays (TTR-31 and receptors), molecular changes at the protein levels, as well as time-lapse assays to distinguish between the role of TTR-31 in triggering germ cell apoptosis or in germ cell corpse clearance should provide novel insights into the functional roles of this TTR-like protein in parasitic nematodes.

In conclusion, we functionally characterised a secretory protein *Hc-*TTR-31 in the important parasitic nematode *H. contortus*. This protein appeared to play roles in regulating post-embryonic larval development, and likely in protecting germ cells from oxidative stress and mediating clearance of apoptotic germ cells. Detailed involvement of *Hc-*TTR-31 in the development and reproduction of *H. contortus* and related parasitic nematodes of socioeconomic importance warrants further investigation. A better understanding of these aspects at the molecular level is likely to indicate potential targets for the control of parasitic diseases.

## Data Availability

The datasets presented in this study can be found in online repositories. The names of the repository/repositories and accession number(s) can be found below: https://www.ncbi.nlm.nih.gov/genbank/, MW013314.
