# Supplementary material for: Haemonchus contortus Transthyretin-Like Protein TTR-31 Plays Roles in Post-Embryonic Larval Development and Potentially Apoptosis of Germ Cells
Source: Front Cell Dev Biol. 2021 Nov 3;9:753667. doi: 10.3389/fcell.2021.753667 (PMC8595280; doi:10.3389/fcell.2021.753667)
Supplement: Supplementary file 1 [file Table1.DOCX]

**Table S1** List of primers used in this study.

| Primers name | Purpose | Sequence (5’-3’) |
| --- | --- | --- |
| *Hc-ttr-31* 1F  *Hc-ttr-31* 1R  *Hc-ttr-31* 2F  *Hc-ttr-31* 2R  *Hc-ttr-31* 3F  *Hc-ttr-31* 3R  *Hc-ttr-31* 4F  *Hc-ttr-31* 4R  *Hc-ttr-31* pF1  *Hc-ttr-31* pR1  *Hc-ttr-31* pF2  *Hc-ttr-31* pR2  *Ce-ttr-31* 1F  *Ce-ttr-31* 1R  *Ce-ttr-31* 2F  *Ce-ttr-31* 2R  *Ce-ttr-31* 3F  *Ce-ttr-31* 3R  *Ce-ttr-31* pF1  *Ce-ttr-31* pR1  *Ce-ttr-31* pF2  *Ce-ttr-31* pR2  *β-tubulin* F  *β-tubulin* R  *act-1* F  *act-1* R  *Ce-ina-1* F  *Ce-ina-1* R  *Ce-egl-1* F  *Ce-egl-1* R  *Ce-ttr-52* F  *Ce-ttr-52* R  *Ce-ced-6* F  *Ce-ced-6* R  *Ce-ced-2* F  *Ce-ced-2* R  *Ce-ced-1* F  *Ce-ced-1* R  *Ce-ced-7* F  *Ce-ced-7* R  *Ce-ced-3* F  *Ce-ced-3* R  *Ce-ced-4* F  *Ce-ced-4* R  *Ce-ced-9* F  *Ce-ced-9* R  *Ce-psr-1* F  *Ce-psr-1* R  *Ce-nrf-5* F  *Ce-nrf-5* R | DNA cloning  DNA cloning  Prokaryotic expression  Prokaryotic expression  RT-PCR  RT-PCR  RNAi  RNAi  Promoter  Promoter  Overexpression  Overexpression  cDNA  cDNA  RT-PCR  RT-PCR  RNAi  RNAi  Promoter  Promoter  Overexpression  Overexpression  RT-PCR  RT-PCR  RT-PCR  RT-PCR  RT-PCR  RT-PCR  RT-PCR  RT-PCR  RT-PCR  RT-PCR  RT-PCR  RT-PCR  RT-PCR  RT-PCR  RT-PCR  RT-PCR  RT-PCR  RT-PCR  RT-PCR  RT-PCR  RT-PCR  RT-PCR  RT-PCR  RT-PCR  RT-PCR  RT-PCR  RT-PCR  RT-PCR | ATGCGGCTTCTGTTAGCTCTTATT  TCAATTGAGACAGTCTCGAGTTTCA  TCCATATGATGCGGCTTCTGTTAGCTCTTATT  CGTCGACTCAATTGAGACAGTCTCGAGTTTCA  TTATTGTCCCCCTGGGTGTCGTTGG  TTTCCTTGAACGGCAGTGCTTTGTA  GGATCCATGCGGCTTCTGTTAGCTCTTATT  AAGCTTTCAATTGAGACAGTCTCGAGTTTCA  TCCCCCCGGGAACCCTCCTGATTCTAACACTATGC  TCCCCCGGGTGAAAAATTAATTTTTTCACCGACG  CGGGATCCATGCGGCTTCTGTTAGCTCTTATT  TGGCCAATTGAGACAGTCTCGAGTTTCACCA  ATGAAATTCGTTTTCATCACATTT  ATTGATACAGTCACGAGACTCTCCT  CTGTATGAGGTCGATCCCATCAAGG  GTCAATTTTGGTCCATTCGGTGTCA  GCTCTAGAATGAAATTCGTTTTCATCACATTT  CCCAAGCTTATTGATACAGTCACGAGACTCTCCT  CCCAAGCTTTTCCTTTTTCTTACTTTCTTTTTTG  CGCGGATCCTCTGAAAAATTGAACATAAGAGCA  CGGGATCCATGAAATTCGTTTTCATCACATTT  TGGCCAATTGATACAGTCACGAGACTCTCCT  GTTGGTAACTCGACTGCTATCC  CTCCATTTCGTCCATACCCTC  GGTTGGTATGGGACAGAAGG  GCTCGTTGTAGAAGGTGTGG  TATGCGAAACGACCCAACGA  GCACACACCAAAGCACTACC  TCAATTTGCCGACGACTCGGG  TCTGTCGGAAGCATGGGCCG  TCGGATGACTTGATGGGTCG  CACGATGTGGGCAATTGTGTT  GGACCTTGGCGGAATCGAAG  TCGGCCAAACCCTGCTAGTAA  TGACGACAAACGGGTTCGAT  ACTTGTGAGCCTCTTCACGG  CCAGCACATGCAGTAACGAC  GTTCGCAGAGTTCTGGGTGA  TACCTCGGCTCTCACACACA  TCATCGGGTTTGGGAAAGAGA  GACATCCCGCCTGCTCAAAA  CGAAAGAGAACTGGGGGAGAA  TTGTGAGCGACACGGATGAC  CAACTGACGTGACATGCTCC  GTCTAATCTCGTTCGGCGGT  CCAGCTCCGATTGTGTTCCT  TGTTGTCGTTTGCCACAGAG  TCTCGCTTTGTCCTTCGCAT  CGCCGAGTTAGGAGCATCAT  TCATCATCCGGTGTTGGAGC |

RT-PCR, SYBR Green real-time PCR; RNAi, RNA interference.
