# Supplementary figures and images for: Haemonchus contortus Transthyretin-Like Protein TTR-31 Plays Roles in Post-Embryonic Larval Development and Potentially Apoptosis of Germ Cells
Source: Front Cell Dev Biol. 2021 Nov 3;9:753667. doi: 10.3389/fcell.2021.753667 (PMC8595280; doi:10.3389/fcell.2021.753667)

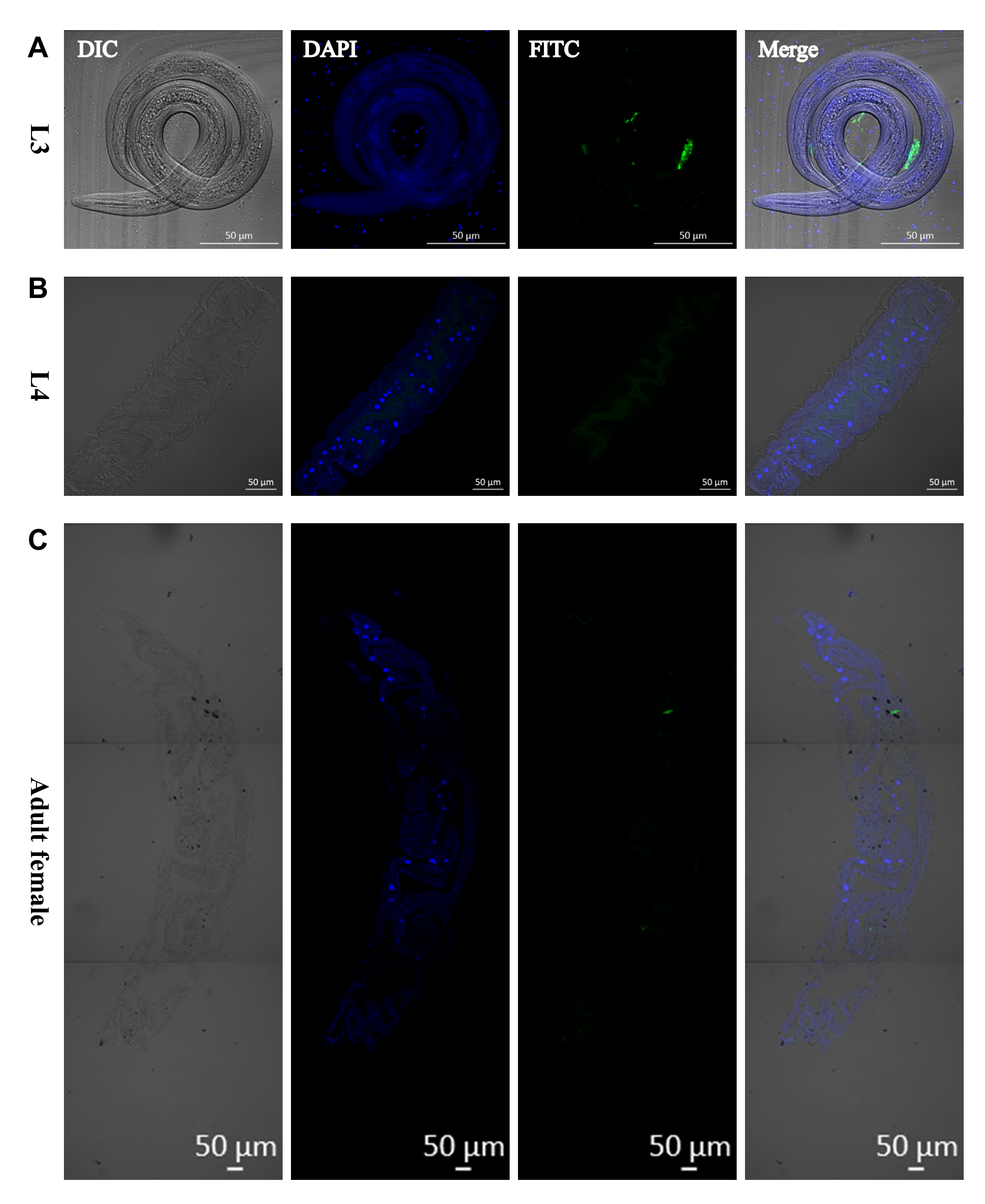

Supplement: Supplementary file 2 [file Image2.TIF]

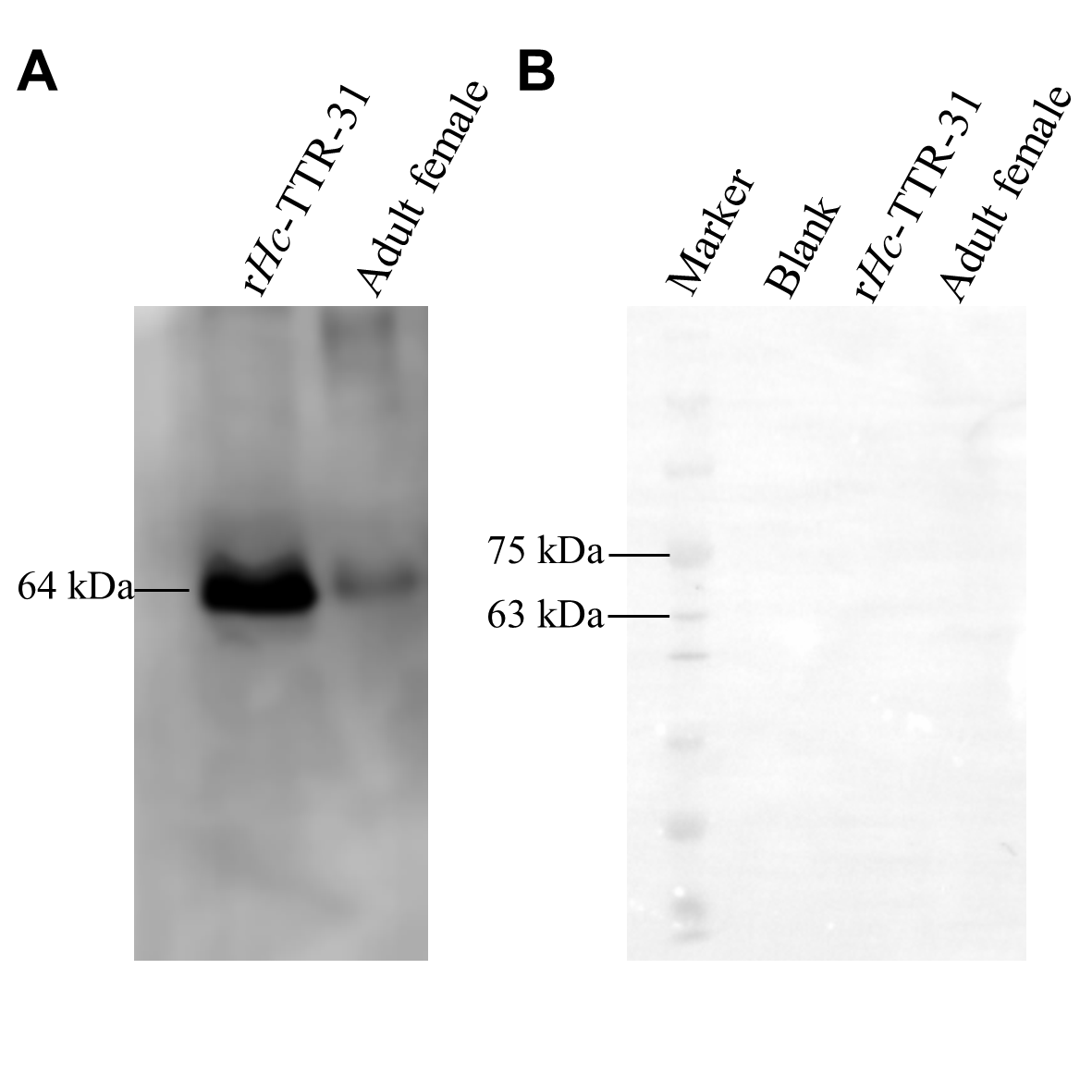

Supplement: Supplementary file 3 [file Image1.TIF]
